# The roles of the general practitioner and sexual health centre in HIV testing: comparative insights and impact on HIV incidence rates in the Rotterdam area, the Netherlands - a cross-sectional population-based study

**DOI:** 10.1186/s12889-023-17483-w

**Published:** 2023-12-21

**Authors:** Denise E. Twisk, Abraham Meima, Jan Hendrik Richardus, Ard van Sighem, Casper Rokx, Jan G. den Hollander, Hannelore M. Götz

**Affiliations:** 1 Department of Public Health, Erasmus MC, University Medical Center Rotterdam, Rotterdam, The Netherlands; 2 Department of Public Health, Municipal Public Health Service Rotterdam-Rijnmond, P.O. Box 70032, Rotterdam, 3000 LP The Netherlands; 3 Department Research and Business Intelligence, Municipality of Rotterdam, Rotterdam, The Netherlands; 4 Centre for Infectious Disease Control, National Institute for Public Health, and the Environment (RIVM), Bilthoven, The Netherlands; 5 Stichting hiv monitoring, Amsterdam, The Netherlands; 6 Department of Internal Medicine, section of infectious diseases, Department of Medical Microbiology and Infectious Diseases, Erasmus University Medical Center, Rotterdam, The Netherlands; 7 Department of Internal Medicine, Maasstad Hospital, Rotterdam, The Netherlands

**Keywords:** HIV, Testing, Primary care, General practitioner, Sexual health centre, Diagnoses, Epidemiology

## Abstract

**Background:**

Access to HIV testing is crucial for detection, linkage to treatment, and prevention. In less urbanised areas, reliance on general practitioners (GPs) for HIV testing is probable, as sexual health centres (SHC) are mostly located within urbanised areas. Limited insight into individuals undergoing HIV testing stems from sparse standard registration of demographics at GPs. This cross-sectional study aims (1) to assess and compare HIV testing at the GP and SHC, and (2) to assess population- and provider-specific HIV incidence.

**Methods:**

Individual HIV testing data of GPs and SHC were linked to population register data (aged ≥ 15 years, Rotterdam area, 2015–2019). We reported the proportion HIV tested, and compared GP and SHC testing rates with negative binomial generalised additive models. Data on new HIV diagnoses (2015–2019) from the Dutch HIV Monitoring Foundation relative to the population were used to assess HIV incidence.

**Results:**

The overall proportion HIV tested was 1.14% for all residents, ranging from 0.41% for ≥ 40-year-olds to 4.70% for Antilleans. The GP testing rate was generally higher than the SHC testing rate with an overall rate ratio (RR) of 1.61 (95% CI: 1.56–1.65), but not for 15-24-year-olds (RR: 0.81, 95% CI: 0.74–0.88). Large differences in HIV testing rate (1.36 to 39.47 per 1,000 residents) and GP-SHC ratio (RR: 0.23 to 7.24) by geographical area were observed. The GPs’ contribution in HIV testing was greater for GP in areas further away from the SHC. In general, population groups that are relatively often tested are also the groups with most diagnoses and highest incidence (e.g., men who have sex with men, non-western). The overall incidence was 10.55 per 100,000 residents, varying from 3.09 for heterosexual men/women to 24.04 for 25–29-year-olds.

**Conclusions:**

GPs have a pivotal role in HIV testing in less urbanised areas further away from the SHC, and among some population groups. A relatively high incidence often follows relatively high testing rates. Opportunities to improve HIV testing have been found for migrants, lower-educated individuals, in areas less urbanised areas and further away from GP/SHC. Strategies include additional targeted testing, via for example SHC branch locations and outreach activities.

**Supplementary Information:**

The online version contains supplementary material available at 10.1186/s12889-023-17483-w.

## Introduction

HIV testing is a first step in the HIV care continuum and depends on accessibility to testing sites. Larger distance to and limited availability of testing sites are associated with lower test rates for a sexually transmitted infection (STI) including HIV [1–3]. Other barriers are concerns about privacy, confidentiality, and stigma [4]. These barriers are possibly greater for non-specialised sexual healthcare settings and people living in low urbanised areas [4, 5]. Public sexual health centres (SHCs) are often located in urban areas. As a result, STI testing in suburban and rural areas likely depends more on other healthcare providers, like general practitioners (GPs).

In the Netherlands, GPs and SHCs are the main two STI care providers. Approximately two-thirds of STI consultations take place in primary care and the rest at the SHC; a minority uses other settings such as private (self-)care [6, 7]. A Dutch study on HIV testing at GP and SHC showed also this 2:1 distribution in the number of HIV tests [8]. GPs test for HIV based on clients’ request or on doctors’ advice. National GP guidelines advise an HIV test for people belonging to key groups (e.g., men who have sex with men (MSM), those form STI/HIV endemic areas, individuals with ≥ 3 sex partners in the last 6 months or those with a partner in one of these groups) and people with HIV indicator conditions [9, 10]. The most relevant HIV indicator conditions for the GP include another STI or hepatitis A/B, herpes zoster, recurrent pneumonia, mononucleosis-like illness, unexplained fever, weight loss, or chronic diarrhoea. An HIV test at the GP may incur costs as part of the compulsory financial contribution for health insurance.

HIV testing is free and anonymous at the SHC. SHCs provide additional STI care, and access is restricted to key populations including those notified for or having symptoms of a STI, MSM, people originating from STI/HIV endemic areas, and sex workers [11]. Accommodating all appointment requests poses a challenge for SHCs, even for individuals belonging to key populations. SHC key populations – except those aged < 25 years who do not belong to another key group – are tested for HIV by default unless they explicitly decline testing (opt-out principle) [11].

In the Netherlands, in line with previous years, most new HIV diagnoses were at the GP (35%) and SHC (30%) in 2020 [12]. The rest is diagnosed at a hospital (29%) or another location (6%) [12]. These percentages represent the initial provider where HIV diagnosis occurs. Signs or symptoms are likely to underlie HIV testing in hospitals after referral by a GP, who acts as gatekeeper to secondary care in the Netherlands. There are approximately 24,000 people with HIV, of whom around 1,600 remain undiagnosed [12]. Over 50% of those diagnosed with HIV are found to have a late-stage infection (CD4 T-cell count < 350 cells/mm^3^ or AIDS-defining event) at time of diagnosis [12]. Most newly diagnosed individuals live in the greater Amsterdam and Rotterdam areas [12]. Compared to Amsterdam, the Rotterdam area has a higher proportion of late-stage infections at diagnosis [12, 13].

Of people with HIV a broad set of characteristics is centrally registered, but there is limited insight in characteristics of people who test for HIV. This is especially due to limited registration of clients’ sociodemographics at the GP, for example migratory background is not registered. At the SHC, sociodemographic and sexual behaviour data of all clients are registered for surveillance purposes. Since access to HIV testing is crucial for early HIV detection, more insight is needed into the people being tested and by which provider. Therefore, we firstly aimed to assess and to compare the sociodemographic characteristics of HIV tested individuals at the GP and SHC. Secondly, we aimed to assess the characteristics of people with HIV relative to the general population. Insight in population- and provider specific HIV testing, and incidence may further aid local HIV testing strategies. We hypothesize large differences in testing, diagnosis, and incidence between subpopulations and by geographical area due to policy and (geographical) differences in access to healthcare providers.

## Methods

We performed a cross-sectional study in the greater Rotterdam area. This study area consists of 15 municipalities segmented into 183 four-digit postal code (PC) areas and harbours around 1.3 million residents (Statistics Netherlands, 2022). PC areas were used as geographical study unit.

### Data sources

#### HIV testing and population data

Individual HIV testing laboratory data of GPs and the central SHC were used (2015–2019). HIV tests for antenatal screening were excluded. The GP data included 12–100% of all general practices within a municipality, with a median coverage of 88% (interquartile range: 60–100%). The municipality with 12% coverage was considered as too low for reliable estimates and therefore excluded. Herewith we excluded around 5% of all residents in the data and the median coverage increased to 90%. SHC data was complete (100% coverage). For each included study year, we stated whether someone was tested for HIV (overall and per provider).

The individual HIV testing data were linked to the population register including all residents within the study area of ≥ 15 years (2015–2019). Population microdata was obtained from the Statistics Netherlands. GP testing data was linked to the population data using a unique anonymous identifier based on citizen service number (98% match). No citizen service number was available in SHC data. We used pseudonymous surveillance data of the SHC, and match these data on a combination of gender, date of birth and PC to the population data (88% match). HIV testing was reported for population records matching HIV testing data (tested), whereas it was not reported for those without a match (not tested).

As a result of HIV testing and population data linkage, the study database included on individual level information on HIV testing by provider (yes/no), sex, age, migratory background (based on country of birth of individual and parents), education level (classification based on International Standard Classification of Education), and distance to the closest general practice from home address. Given a 36% lack of education level data and assuming non-random missingness for individuals over 60 years due to national registration gaps, we used multiple imputation via chained equations (n = 5 imputed datasets with each 10 iterations) to impute education level for < 60-year-olds. As a result, only 15% of the individuals had missing information on education level. Also, information on PC level was available: urbanisation level and median income per household as indicator for area socio-economic status. Additionally, straight-line distance from PC centroid to SHC was linked to the database [2].

#### HIV data

HIV treatment centres provide care to people with HIV diagnosed at GP, SHC, hospitals and other test settings. Other test settings include a diagnosis abroad, at another location (e.g., antenatal HIV testing, rapid testing at NGO healthcare facility, a self-test, medical examination) or if diagnosis location is unknown. For purpose of HIV monitoring, surveillance, and research, pseudonymised patient data from HIV treatment centres are centrally collected in the ATHENA national HIV cohort at stichting hiv monitoring (SHM; HIV Monitoring Foundation). In contrast to SHM data, which includes people diagnosed at all possible locations, our HIV testing data is limited to diagnoses made at GP and SHC. Therefore, we used SHM data on new HIV diagnoses (2015–2019) of people aged ≥ 15 years living within the study area to assess HIV incidence. Hence, the SHM database includes partly the same individuals as the laboratory HIV testing databases. The SHM database was not linked to the population database, unlike the linkage established for HIV testing data. SHM collects data of all people that receive HIV care in one of the 24 HIV treatments centres in the Netherlands: location of diagnosis, PC at entry of care, the demographics sex, country of birth, age, transmission mode, and clinical and virological data. Based on the PC at entry into care, we enriched the SHM database with publicly available PC level data of Statistics Netherlands and straight-line distance from PC centroid to SHC [2, 14]. This study is limited to 98% of all people with HIV within the study age and area, because we did not receive consent from all HIV treatment centres.

### Statistical analysis

We reported the socio-demographic and PC level characteristics of individuals tested for HIV. Subsequently, the mean HIV testing rates (number of HIV tests per 1,000 residents) over the study period were compared between GP and SHC per subpopulation and PC area. The HIV testing rates were corrected for incomplete HIV testing data. SHC numbers were corrected with 100/88, considering the 88% match between SHC and population data. If, for example, 1000 tests were initially reported, the correction would be: 1000 * (100/88) = 1136, offering a more accurate representation of the actual HIV testing numbers. GP numbers were corrected by 100 divided by the municipality-specific GP laboratory data coverage. Numbers and rates were based on 5-year counts (2015–2019) to mitigate analytical problems caused by small numbers of cases per subpopulation or PC area, and to preserve anonymity of the cases. GP and SHC testing rates were compared using generalised additive models (GAM) with a negative binomial distribution calculating rate ratios (RR) and their 95% confidence intervals (CI). In these models, SHC was used as reference and the log of the total number of residents as offset. Finally, we assessed the characteristics of diagnosed individuals – relative to the population – that were available from the population and SHM databases. We did not report on subpopulations or areas with less than 10 cases to maintain anonymity. The minimum of 10 cases applies to both the numerator and denominator. We used R version 3.6.2 for analyses and to create geographical plots.

## Results

### Characteristics of tested population

Characteristics of the general population and the HIV tested population are presented in Table 1 and in more detail in Supplementary Table 1. The proportion HIV tested was 1.14% for all residents and up to 4.70% for Antilleans. Antilleans were most tested at both GP (2.97%) and SHC (1.86%). Those tested least were older age groups and people from less urbanised areas, at both providers. Over the studied years, the number of residents slightly increased, but the number of tested individuals decreased (Supplementary Table 1). This was mainly caused by a decrease in the number of individuals tested by the SHC. In total, 19.96% of the SHC clients were tested more than once within the study period, while this was 5.66% for GP clients.

**Table 1:** Characteristics of general and HIV tested population, and GP-SHC comparison of HIV testing rates, 2015–2019^1^

	General population	Tested^2^	Tested by GP^2^	Tested by SHC^2^	Mean HIV testing rates GP vs. SHC^2^
	No (%)	No (%; row%)	No (%; row%)	No (%; row%)	RR (95% CI)^3^
**Total**	5,107,921 (100.00%)	58,356 (100.00%; 1.14%)	37,150 (100.00%; 0.73%)	22,394 (100.00%; 0.44%)	1.61 (1.56–1.65)
Mean per year	1,021,584	11,671	7430	4479	
**Individual**					
**Sex**					
Men	2,490,618 (48.76%)	30,856 (52.88%; 1.24%)	18,082 (48.67%; 0.73%)	13,516 (60.36%; 0.54%)	1.30 (1.24–1.35)
Women	2,617,303 (51.24%)	27,500 (47.12%; 1.05%)	19,068 (51.33%; 0.73%)	8878 (39.64%; 0.34%)	2.08 (2.02–2.14)
**Age (in years)**					
15–24	758,131 (14.84%)	16,117 (27.62%; 2.13%)	7526 (20.26%; 0.99%)	8960 (40.01%; 1.18%)	0.81 (0.74–0.88)
25–29	445,054 (8.71%)	14,222 (24.37%; 3.20%)	8338 (22.44%; 1.87%)	6215 (27.75%; 1.40%)	1.30 (1.22–1.37)
30–34	423,086 (8.28%)	9378 (16.07%; 2.22%)	6704 (18.05%; 1.58%)	2867 (12.80%; 0.68%)	2.27 (2.17–2.36)
35–39	396,052 (7.75%)	5964 (10.22%; 1.51%)	4487 (12.08%; 1.13%)	1580 (7.06%; 0.40%)	2.75 (2.62–2.87)
≥ 40	3,085,598 (60.41%)	12,675 (21.72%; 0.41%)	10,095 (27.17%; 0.33%)	2772 (12.38%; 0.09%)	3.53 (3.43–3.62)
**Migratory background**					
Native Dutch	3,260,384 (63.83%)	24,559 (42.08%;0.75%)	15,828 (42.61%; 0.49%)	9174 (40.97%; 0.28%)	1.67 (1.61–1.73)
Western	555,326 (10.87%)	6682 (11.45%; 1.20%)	4214 (11.34%; 0.76%)	2595 (11.59%; 0.47%)	1.57 (1.46–1.68)
Middle and Eastern European	180,872 (3.54%)	1983 (3.40%; 1.10%)	1248 (3.36%; 0.69%)	789 (3.52%; 0.44%)	1.53 (1.34–1.73)
Other Western	374,454 (7.33%)	4699 (8.05%; 1.25%)	2966 (7.98%; 0.79%)	1806 (8.06%; 0.48%)	1.56 (1.38–1.74)
Non-Western	1,292,211 (25.30%)	27,115 (46.46%;2.10%)	17,108 (46.05%; 1.32%)	10,625 (47.45%; 0.82%)	1.56 (1.50–1.61)
Dutch Antillean	132,989 (2.60%)	6253 (10.72%; 4.70%)	3956 (10.65%; 2.97%)	2474 (11.05%; 1.86%)	1.55 (1.43–1.66)
Surinamese	305,053 (5.97%)	7140 (12.24%; 2.34%)	4551 (12.25%; 1.49%)	2762 (12.33%; 0.91%)	1.59 (1.49–1.70)
Turkish	260,436 (5.10%)	2436 (4.17%; 0.94%)	1581 (4.26%; 0.61%)	900 (4.02%; 0.35%)	1.70 (1.52–1.88)
Moroccan	189,405 (3.71%)	2518 (4.31%; 1.33%)	1600 (4.31%; 0.84%)	969 (4.33%; 0.51%)	1.59 (1.42–1.77)
Other non-Western	266,458 (5.22%)	4770 (8.17%; 1.79%)	2853 (7.68%; 1.07%)	2009 (8.97%; 0.75%)	1.38 (1.25–1.51)
Sub-Saharan African^4^	57,714 (1.13%)	1640 (2.81%; 2.84%)	1073 (2.89%; 1.86%)	589 (2.63%; 1.02%)	1.77 (1.55–1.98)
Cape Verdean	80,156 (1.57%)	2358 (4.04%; 2.94%)	1494 (4.02%; 1.86%)	922 (4.12%; 1.15%)	1.56 (1.38–1.74)
**Education level (imputed)** ^**5,6**^					
Low	1,420,610 (32.98%)	1,420,610 (27.88%; 1.14%)	11,056 (30.06%; 0.78%)	5376 (24.14%; 0.38%)	1.99 (1.92–2.07)
Medium	1,724,767 (40.04%)	1,724,767 (46.37%; 1.56%)	16,451 (44.73%; 0.95%)	10,962 (49.22%; 0.64%)	1.45 (1.40–1.51)
High	1,162,096 (26.98%)	1,162,096 (25.76%; 1.28%)	9269 (25.20%; 0.80%)	5934 (26.64%; 0.51%)	1.51 (1.44–1.58)
Missing	800,448	482	374	122	
**Area**					
**Municipality**					
Municipality of Rotterdam	2,657,426 (52.03%)	44,685 (76.57%; 1.68%)	27,971 (75.29%; 1.05%)	17,694 (79.01%; 0.67%)	1.52 (1.47–1.56)
Surrounding municipalities	2,450,495 (47.97%)	13,671 (23.43%; 0.56%)	9179 (24.71%; 0.37%)	4700 (20.99%; 0.19%)	1.94 (1.86–2.02)
**Degree of urbanisation**					
Very high (≥ 2500 addresses/km^2^)	2,414,666 (47.29%)	41,598 (71.30%; 1.72%)	25,382 (68.34%; 1.05%)	17,147 (76.58%; 0.71%)	1.43 (1.38–1.47)
Other (< 2500 addresses/km^2^)	2,691,627 (52.71%)	16,745 (28.70%; 0.62%)	11,759 (31.66%; 0.44%)	5243 (23.42%; 0.19%)	2.20 (2.12–2.27)
Missing	1628	13	9	4	
**Median household income**					
Highest (>€36,600)	1,155,750 (22.64%)	6304 (10.81%; 0.55%)	4402 (11.85%; 0.38%)	1990 (8.89%; 0.17%)	2.10 (1.98–2.21)
Upper middle (€28,400 - €36,600)	74,093 (1.45%)	672 (1.15%; 0.91%)	387 (1.04%; 0.52%)	293 (1.31%; 0.40%)	1.24 (0.92–1.57)
Other (<€28,200)	3,876,135 (75.91%)	51,365 (88.04%;1.33%)	32,351 (87.11%; 0.85%)	20,106 (89.80%; 0.52%)	1.56 (1.52–1.61)
Missing	1943	15	10	5	
**Distance to closest general practice (in km)** ^**7**^					
< 1	3,864,777 (75.87%)	49,550 (86.08%; 1.28%)	31,336 (85.26%; 0.81%)	19,250 (87.58%; 0.50%)	1.57 (1.52–1.61)
≥ 1	1,229,173 (24.13%)	8012 (13.92%; 0.65%)	5417 (14.74%; 0.44%)	2731 (12.42%; 0.22%)	1.98 (1.88–2.08)
Missing	13,971	794	397	413	
**Distance to SHC (in km)**					
< 5	1,832,795 (35.89%)	35,399 (60.67%; 1.93%)	21,112 (56.84%; 1.15%)	15,103 (67.45%; 0.82%)	1.34 (1.29–1.39)
5–10	1,955,923 (38.30%)	16,757 (28.72%; 0.86%)	11,732 (31.59%; 0.60%)	5312 (23.72%; 0.27%)	2.20 (2.13–2.27)
> 10	1,317,575 (25.80%)	6187 (10.60%; 0.47%)	4297 (11.57%; 0.33%)	1975 (8.82%; 0.15%)	2.02 (1.90–2.13)
Missing	1628	13	9	4	

Abbreviations: CI, confidence interval; GP, general practitioner; km, kilometre; no, number; RR, rate ratio; SHC, sexual health centre

^1^ The data underlying this table are the GP and SHC laboratory data, and the population register data (2015–2019)

^2^ Proportion tested is based on the raw numbers. The mean HIV testing rates (number of HIV tests per 1,000 residents) are calculated over the study period of 5 year and corrected for data incompleteness. The number of tests by SHC was corrected with 1/0.88, considering the 88% match between SHC and population data. For each municipality, the number of tests by the GP was corrected by 1/coverage (municipality specific). The corrected SHC numbers are on average 13% higher (min. 9% - max. 15%), and the corrected GP numbers on average 9% higher (min. 4% - max. 13%)

^3^ HIV testing rate comparison, with SHC as reference

^4^ Without Cape Verdean

^5^ The International Standard Classification of Education was used as basis (European Commission; available from: https://ec.europa.eu/eurostat/statistics-explained/index.php?title=International_Standard_Classification_of_Education_(ISCED)#:~:text=ISCED%201%3A%20Primary%20education,Post%2Dsecondary%20non%2Dtertiary%20education)

^6^ Multiple imputation via chained equations (MICE) using ten iterations of five multiple imputations. Only imputed for < 60 years-old, above 60 years missingness was assumed not at random due to absence of national registration

^7^ Based on address of residential location. Other area characteristics are based on the 4-digit postal code of residential location

### Comparing GP and SHC testing rate by subpopulation

Overall, the HIV testing rate was 1.61 times higher for GPs than for the SHC (95%CI: 1.56–1.65; Table 1). Only individuals < 25 years had a lower testing rate at the GP compared to the SHC (RR: 0.81, 95%CI: 0.74–0.88; Table 1). This was mainly driven by 20–24-year-olds (RR: 0.74, 95%CI 0.66–0.82) than by 15–19-year-olds (RR: 1.11, 95%CI: 1.97–1.25; Supplementary Table 1), and independently of western or non-western background (Supplementary Table 1). Despite of large differences in proportion tested, migrant groups did not differ substantially in GP-SHC ratio (RR ranges from 1.38 to 1.77). The test contribution of GPs was greater for lower educated (compared to medium/higher educated), in less urbanised areas (compared to very high urbanised), in areas with the highest median household income (compared to lower incomes) and in areas where distances to GP and SHC are larger (compared to smaller distances). Over time, the HIV testing rate at the GP increased compared to the SHC; from 1.5 times higher in 2015 (RR: 1.52, 95%CI: 1.43–1.60) to almost 2 times higher in 2019 (RR: 1.96, 95%CI: 1.87–2.04) (Supplementary Table 1).

### Comparing GP and SHC testing rate by geographical area

**Fig. 1:**
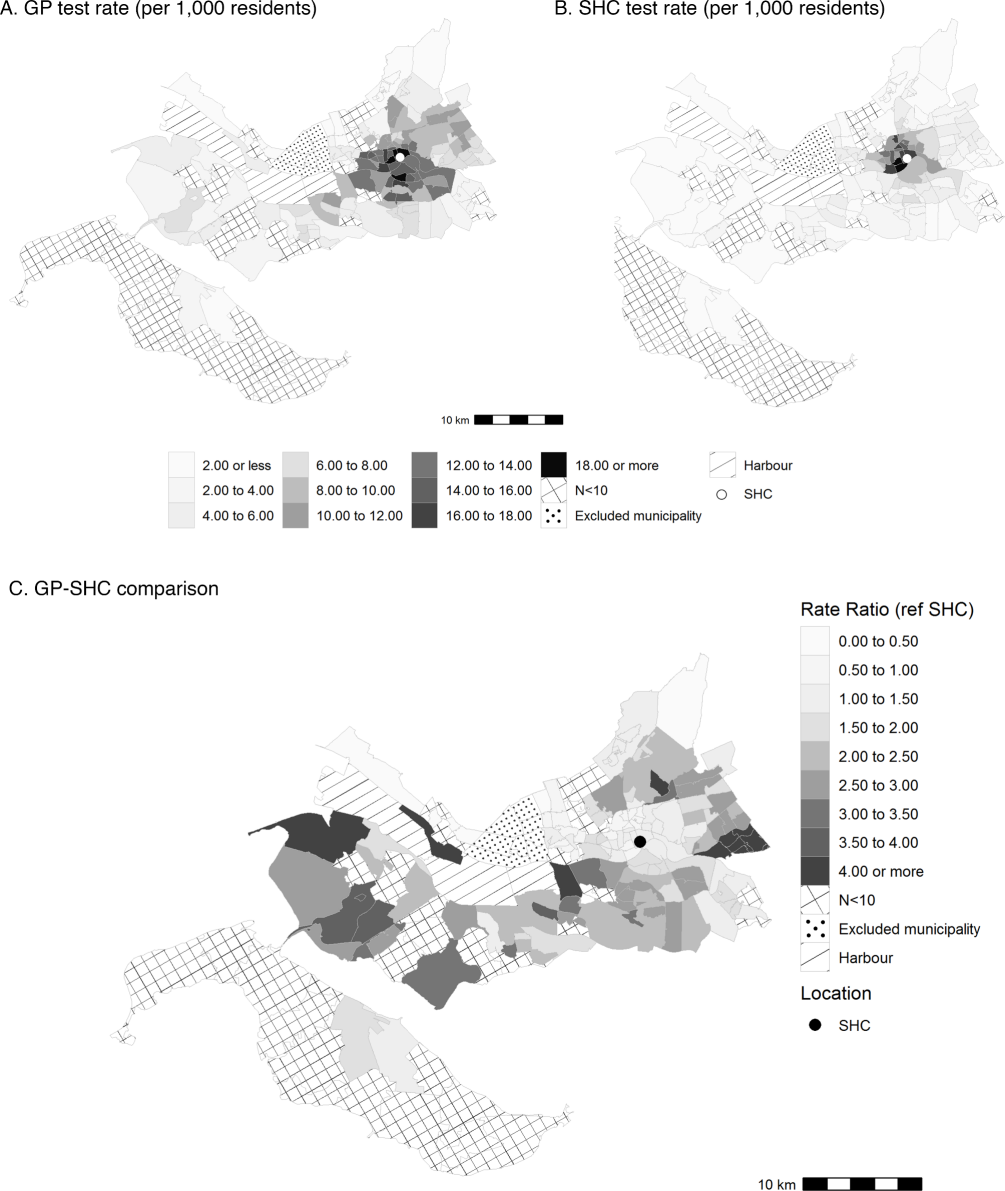
Provider-specific HIV testing rate and GP-SHC testing rate comparison by postal code area, 2015–2019. Abbreviations: GP, general practitioner; N, number; ref, reference; SHC, sexual health centre

We also examined provider-specific HIV testing rates (Fig. 1A and 1B) and GP-SHC test ratio by PC area (Fig. 1C). In total 20% of the PC areas had to be excluded due to less than 10 HIV tests at the GP and/or SHC. Large differences in the HIV testing rate were observed for the remaining areas, ranging from 1.36 to 39.47 per 1,000 residents. Stratified by provider, the HIV testing rate per 1,000 residents ranged from 0.25 to 19.23 for GP and from 0.78 to 20.63 for SHC. The highest SHC testing rates were clustered around the SHC location in the northern part of the area (Fig. 1A). The testing rate at the GP was geographically more widespread compared to the SHC (Fig. 1B The GP-SHC ratio ranged from 0.23 (95%CI: 0.00-0.89) to 7.24 (95%CI: 6.82–7.67). The ratio was lowest in the inner-city of Rotterdam where also the SHC is located. The contribution of GPs in HIV testing was greater in more remote areas.

### People with HIV and HIV incidence

Of the 539 people diagnosed between 2015 and 2019 in the SHM database, 28.94% was diagnosed at the GP, 28.57% in a hospital, 26.90% at the SHC, 6.10% abroad, 5.40% at another location and for 4.10% setting of diagnosis was unknown. The number of diagnoses and incidence varied largely between different demographic and area characteristics (Table 2). However, largely the same patterns were observed between healthcare providers, such as most diagnoses among MSM and people from very urbanised areas. This was not the case for age; hospitals diagnose most people above the age of 40, while other providers diagnose mainly younger people, peaking at the age group 25–29 years. The overall incidence was 10.55 per 100,000 residents (range 3.09 to 24.04). In general, groups that are relatively often tested such as younger age, non-western migratory background, living in urban areas and closely to GP or SHC (Table 1), also have a higher number of diagnoses and incidence (Table 2). Comparing GP and SHC incidences, GPs more frequently diagnose heterosexual men and women, older individuals, residents outside urban areas and those in regions with higher median household income.

**Table 2:** Providers-specific HIV diagnoses and incidence per 100,000 residents, 2015–2019^1^

	Total		GP		SHC		Hospital		Other^2^	
	No. (%)	Incidence	No. (%)	Incidence	No. (%)	Incidence	No. (%)	Incidence	No. (%)	Incidence
**Total**	539	10.55	156	3.05	145	2.84	154	3.01	84	1.64
**Individual**										
**Sex/transmission group**										
MSM	349 (68.80%)	14.01	104 (68.90%)	4.18	118 (86.80%)	4.74	74 (53.20%)	2.97	53 (65.40%)	2.13
Heterosexual men and women	158 (31.20%)	3.09	47 (31.10%)	0.92	18 (13.20%)	0.35	65 (46.80%)	1.27	28 (34.60%)	0.55
Missing	32		5		9		15		3	
**Age at diagnosis (in years)**										
15–24	83 (15.40%)	10.95	19 (12.20%)	2.51	29 (20.00%)	3.83	22 (14.30%)	2.90	13 (15.50%)	1.71
25–29	107 (19.90%)	24.04	29 (18.60%)	6.52	39 (26.90%)	8.76	14 (9.10%)	3.15	25 (29.80%)	5.62
30–34	79 (14.70%)	18.67	26 (16.70%)	6.15	20 (13.80%)	4.73	17 (11.00%)	4.02	16 (19.00%)	3.78
35–39	60 (11.10%)	15.15	12 (7.70%)	3.03	20 (13.80%)	5.05	15 (9.70%)	3.79	13 (15.50%)	3.28
≥ 40	210 (39.00%)	6.81	70 (44.90%)	2.27	37 (25.50%)	1.20	86 (55.80%)	2.79	17 (20.20%)	0.55
**Migratory background**										
Native Dutch	267 (49.50%)	8.19	86 (55.10%)	2.64	78 (53.80%)	2.39	81 (52.60%)	2.48	22 (26.20%)	0.67
Western	57 (10.60%)	10.26	14 (9.00%)	2.52	12 (8.30%)	2.16	11 (7.10%)	1.98	20 (23.80%)	3.60
Non-Western	215 (39.90%)	16.64	56 (35.90%)	4.33	55 (37.90%)	4.26	62 (40.30%)	4.80	42 (50.00%)	3.25
**Area**										
**Municipality**										
Municipality of Rotterdam	398 (74.10%)	14.98	105 (67.30%)	3.95	120 (82.80%)	4.52	109 (71.20%)	4.10	64 (77.10%)	2.41
Surrounding municipalities	139 (25.90%)	5.67	51 (32.70%)	2.08	25 (17.20%)	1.02	44 (28.80%)	1.80	19 (22.90%)	0.78
Missing	2		0		0		1		1	
**Degree of urbanisation**										
Very high (≥ 2500 addresses/km^2^)	403 (75.00%)	16.69	111 (71.20%)	4.60	118 (81.40%)	4.89	112 (73.20%)	4.64	62 (74.70%)	2.57
Other (< 2500 addresses/km^2^)	134 (25.00%)	4.98	45 (28.80%)	1.67	27 (18.60%)	1.00	41 (26.80%)	1.52	21 (25.30%)	0.78
Missing	2		0		0		1		1	
**Median household income**										
Highest and upper middle (≥€28.400)	199 (37.10%)	16.18	72 (46.20%)	5.85	36 (24.80%)	2.93	53 (34.90%)	4.31	38 (45.80%)	3.09
Other (<€28.400)	337 (62.90%)	8.69	84 (53.80%)	2.17	109 (75.20%)	2.81	99 (65.10%)	2.55	45 (54.20%)	1.16
Missing	3		0		0		2		1	
**Distance to closest general practice (in km)** ^**3**^										
< 1	489 (91.10%)	12.65	143 (91.70%)	3.70	135 (93.10%)	3.49	-	-	-	-
≥ 1	48 (8.90%)	3.91	13 (8.30%)	1.06	10 (6.90%)	0.81	-	-	-	-
Missing	2		0		0		1		1	
**Distance to SHC (in km)** ^**3**^										
< 5	292 (54.40%)	15.93	75 (48.10%)	4.09	97 (66.90%)	5.29	-	-	-	-
5–10	188 (35.00%)	9.61	66 (42.30%)	3.37	36 (24.80%)	1.84	-	-	-	-
> 10	57 (10.60%)	4.33	15 (9.60%)	1.14	12 (8.30%)	0.91	-	-	-	-
Missing	2		0		0		1		1	

Abbreviations: GP, general practitioner; km, kilometre; MSM; men who have sex with men, no, number; SHC, sexual health centre

^1^ The data underlying this table are the SHM database which includes all people living with HIV that receive care, and the population register data for incidence estimates (2015–2019)

^2^ The provider group Other consists of people diagnosed abroad, diagnosed at another location and diagnosed at an unknown location

^3^ Data for provider group Hospital and Other not shown because of low number of diagnoses by the provider group Other for one distance category (N < 10)

## Discussion

In this cross-sectional population-based study we found large differences between subpopulations tested for HIV, and testing rates and incidence by subpopulation and geographical area. Generally, the subpopulation-specific HIV testing rate was higher for GPs than for the SHC. However, large differences were observed geographically with areas relatively close to the SHC mainly served by the SHC rather than GPs. HIV incidence was highest for men (MSM), younger age groups, non-western people and people residing in urban areas close to primary healthcare providers. For most population groups a relatively high number of diagnoses and incidence follows relatively high testing rates.

The proportion tested of the general population in the Netherlands within the study period was limited to 1.14%. No other studies reported estimates for this HIV test proportion. This proportion is substantially different compared to the 3% for chlamydia and gonorrhoea testing, which we estimated in a previous study with the same design, timespan, and study area [15]. The same discrepancy is noted for key populations recommended for HIV testing, such as people with a non-western migratory background. This indicates missed opportunities for HIV testing and provides opportunities for improvement. National STI consultation guidelines recommend pre-emptive testing for multiple STIs, including HIV, for key populations and HIV testing in all with a proven STI [9–11]. Previous research also showed that GPs follow these guidelines to a limited extent [16–18].

In addition to an “offer everything” strategy for key populations, HIV testing based on HIV indicator condition is pivotal for the detection of undiagnosed HIV cases. Since 99% of the Dutch population is registered at a general practice and 75% of the population contacts the GP at least once per year, the GP is an important provider in HIV indicator guided testing. HIV testing based on HIV indicator conditions at the GP is – apart from key populations – crucial and cost-effective for people not typically considered at risk for HIV, such as women and heterosexual (older) people [9, 10]. The key role of GPs for these groups is also corroborated by our study as we found that people with HIV diagnosed at the GP were more likely to be female, heterosexual males and older compared to the SHC. These findings are in line with a previous nationwide Dutch study [17]. GP educational meetings including reviewing guideline compliance (e.g., “offer everything” strategy for key populations and HIV indicator condition guided testing for all) could increase awareness, confidence, and consideration of HIV testing [19].

In our study we also found opportunities for improved HIV testing for specific groups. Specific activities by the SHC to reach people with a lower education, people from less urbanised areas and people living more distant from testing sites may be considered. This was also observed in our study on chlamydia and gonorrhoea testing, in which we additionally found that lower education, urbanisation and distance to testing site were independently associated with testing [15]. We observed that these groups were underrepresented within the HIV tested population. Although almost all subpopulations are tested more by the GP than by the SHC, the contribution of the GP in the aforementioned groups is even higher. Possible explanations are that lower educated are unaware of the SHC testing services, and reduced SHC accessibility for people from less urbanised areas, which are usually also the areas further away from the testing sites. Our GP-SHC testing rate comparison by geographical area is in line with the latter: the SHC seems the dominant test provider in areas closely to the SHC, while the GP takes on this role in areas more distant from the SHC. A branch SHC in less urbanised areas and/or more outreach testing or remote testing may be considered [20]. Outreach activities, for example at community-based organisations, are also likely to reach migrant people better. Migrant people are an important key group for improved HIV testing, as they are more often diagnosed with a late-stage HIV infection [13, 21], and generally face more barriers to test [22–24].

The overall HIV incidence (2015–2019) of 10.55 per 100,000 residents in our study area is more than twice as high as the estimated nationwide incidence (4.3 per 100,000) [12]. Although, the incidence differs largely between subgroups, the observed pattern is in line with our expectations and corresponds with other literature, for example the highest incidence for 25–29 year-olds [25]. We only have information about HIV testing at GP and SHC, but we showed that generally more HIV testing is followed by a relatively high number of diagnoses and incidences. More HIV testing is likely related to easier access to testing services due to priority in policy and guidelines (for younger age groups, men (MSM), and non-western people) and the convenience of proximity (for people living more urban and closer to healthcare providers). A low testing rate was for example observed for people above 40 years, while there were a high number of new HIV cases in this group. A possible explanation is selective testing in both instances. The high number of cases may have been detected by clinical criteria for testing [26]. On the other hand, low testing rates may be due to low-risk perception (patient and provider), low awareness of HIV testing because of low HIV prevalence in non-key populations (provider), and miss preconceptions about sexuality and HIV risk for older people (provider) [26, 27].

### Strengths and limitations

The strength of this study is that we linked population and laboratory data of the two main STI test providers. Herewith we ruled out responder, recall and registration biases associated with for example questionnaires [18, 28]. Further, we are the first that provide a unique and comprehensive assessment of characteristics of HIV tested individuals. Our study has some limitations. First, we were not able to include all laboratory testing data of GPs and SHC data in our study area. To limit the effect of this, we corrected our testing rates and GP-SHC comparison for incomplete data. An examination of SHC registered characteristics revealed that non-matched individuals were more frequently tested for HIV [15]. This might have influenced the observed differences in testing rates between GP and SHC but not the direction of the effect. Second, the current study is limited to GPs and the SHC and has no information about HIV tests performed via other providers or online testing services. However, GPs and SHC are the main STI test providers and additionally GPs have a gatekeeper role in referring to the hospital [6]. Consequently, most opportunities for improving HIV testing are probably at the GP and SHC. Third, the findings in our study may differ to other parts of the country and other countries and should therefore be generalised with caution. However, our study design can be applied anywhere if population microdata and individual HIV testing data are available. Fourth, the current study lacks detailed information about motives and barriers for HIV testing (e.g., presence of symptoms, notification by sex partner), both from client and provider perspective. Also extra GP client characteristics, for example whether someone is MSM, could provide valuable insight as MSM are advised to test regularly and most newly-diagnosed infections occur among MSM. However, clients’ characteristics are not shared with laboratories, only registered to a limited degree in GP electronic medical records and unknown at population level [29]. Fifth, not all people with HIV diagnosed within the study period are included in the SHM data used in this study: (1) 1.1% opt-out to share their data with SHM, (2) we lack consent from all HIV treatment centres (~ 2% of the cases) and (3) possibly not all people with HIV in care are yet registered at SHM. Although the magnitude of diagnoses and incidence may change when including all cases, we do not expect that the direction of the findings differ as they are in line with previous studies [12, 17, 25]. Finally, for the HIV incidence estimates we were restricted to a limited set of population groups due to small numbers and because of limitations in available information in the SHM database. More accurate and additional insights in individual characteristics of people with HIV might be obtained by extending the number of study years, and by matching the SHM database to population microdata too.

## Conclusions

Our findings show that GPs are the main HIV testing provider. They are especially important in less urbanised areas further away from the SHC. As new diagnoses and the proportion of people with HIV still undiagnosed are getting smaller, adherence to guidelines is important (e.g., HIV testing of key populations during STI consultation and HIV indicator guided testing). Educational meetings or other proactive interventions should encourage GPs (and other physicians) to follow test guidelines and share responsivity in the fight against HIV. Additional testing services for example via SHC branch locations and outreach activities are promising to increase (geographical) access and to test people who are usually not tested at the GP and SHC. HIV test providers, policy makers and communities are urged to collaborate to achieve this. Expanding the provision of HIV testing should be monitored to investigate whether it contributes to new diagnoses.

### Electronic supplementary material

Below is the link to the electronic supplementary material.

**Supplementary Material 1**: **Supplementary Table 1**. Additional characteristics of general and HIV tested population, and GP-SHC comparison of HIV testing rates, 2015–2019. **Supplementary Table 2**. Providers-specific HIV diagnoses and incidence per 100,000 residents by year, 2015–2019

## Data Availability

The datasets generated and/or analysed during the current study are not publicly available due to legal restrictions and privacy restrictions. HIV testing data (de-identified and without linkage to population data) is available upon reasonable request from the corresponding author. Nonpublic population microdata is accessible for statistical and scientific research by authorised institutions at Statistic Netherlands (fees apply). Procedures can be found at https://www.cbs.nl/; for further information, email microdata@cbs.nl. Data on HIV diagnoses from stichting hiv monitoring (SHM; HIV Monitoring Foundation) is available upon reasonable request at SHM. Requests for data access can be made to: hiv.monitoring@amsterdamumc.nl. These will be reviewed on a case-by-case basis, given that the data underlying this study contain sensitive information.

## List of abbreviations

AIDS: Acquired immunodeficiency syndrome
CD4: Cluster of differentiation 4
CI: Confidence interval
GAM: Generalised additive models
GP: General practitioners
HIV: Human immunodeficiency virus
Km: kilometre
MSM: Men who have sex with men
No: Number
PC: Postal code
REF: Reference
RR: Rate ratio
SHC: Sexual health centre
SHM: Stichting hiv monitoring (HIV Monitoring Foundation)
STI: Sexually transmitted infection
