# Supplementary material for: The roles of the general practitioner and sexual health centre in HIV testing: comparative insights and impact on HIV incidence rates in the Rotterdam area, the Netherlands - a cross-sectional population-based study
Source: BMC Public Health. 2023 Dec 21;23:2553. doi: 10.1186/s12889-023-17483-w (PMC10734097; doi:10.1186/s12889-023-17483-w)
Supplement: Supplementary file 1 — Supplementary Material 1: Supplementary Table 1. Additional characteristics of general and HIV tested population, and GP-SHC comparison of HIV testing rates, 2015–2019. Supplementary Table 2. Providers-specific HIV diagnoses and incidence per 100,000 residents by year, 2015–2019 [file 12889_2023_17483_MOESM1_ESM.pdf]

Appendix – Supplementary Tables

Supplementary Table 1. Additional characteristics of general and HIV tested population, and GP-SHC comparison of HIV testing rates, 2015-2019[1]

	General population	Tested[2]	Tested by GP[2]	Tested by SHC[2]	Mean HIV testing rates GP vs. SHC[2]
	No (%)	No (%; row%)	No (%; row%)	No (%; row%)	RR (95% CI)[3]
**Total**	5107921 (100.00%)	58356 (100.00%; 1.14%)	37150 (100.00%; 0.73%)	22394 (100.00%; 0.44%)	1.61 (1.56 - 1.65)
2015	1005596 (19.69%)	11810 (20.24%; 1.17%)	7348 (19.78%; 0.73%)	4693 (20.96%; 0.47%)	1.52 (1.43 - 1.60)
2016	1012665 (19.83%)	11862 (20.33%; 1.17%)	7343 (19.77%; 0.73%)	4753 (21.22%; 0.47%)	1.50 (1.41 - 1.58)
2017	1021033 (19.99%)	12036 (20.63%; 1.18%)	7370 (19.84%; 0.72%)	4899 (21.88%; 0.48%)	1.46 (1.37 - 1.54)
2018	1029952 (20.16%)	11571 (19.83%; 1.12%)	7514 (20.23%; 0.73%)	4301 (19.21%; 0.42%)	1.69 (1.61 - 1.78)
2019	1038675 (20.33%)	11077 (18.98%; 1.07%)	7575 (20.39%; 0.73%)	3748 (16.74%; 0.36%)	1.96 (1.87 - 2.04)
**Individual**					
**Age (in years)**					
15-19	350154 (6.86%)	3602 (6.17%; 1.03%)	1961 (5.28%; 0.56%)	1718 (7.67%; 0.49%)	1.11 (0.97 - 1.25)
20-24	407977 (7.99%)	12515 (21.45%; 3.07%)	5565 (14.98%; 1.36%)	7242 (32.34%; 1.78%)	0.74 (0.66 - 0.82)
25-29	445054 (8.71%)	14222 (24.37%; 3.20%)	8338 (22.44%; 1.87%)	6215 (27.75%; 1.40%)	1.30 (1.22 - 1.37)
30-34	423086 (8.28%)	9378 (16.07%; 2.22%)	6704 (18.05%; 1.58%)	2867 (12.80%; 0.68%)	2.27 (2.17 - 2.36)
35-39	396052 (7.75%)	5964 (10.22%; 1.51%)	4487 (12.08%; 1.13%)	1580 (7.06%; 0.40%)	2.75 (2.62 - 2.87)
40-44	398681 (7.81%)	4115 (7.05%; 1.03%)	3287 (8.85%; 0.82%)	905 (4.04%; 0.23%)	3.52 (3.36 - 3.68)
45-49	440438 (8.62%)	3132 (5.37%; 0.71%)	2534 (6.82%; 0.58%)	643 (2.87%; 0.15%)	3.80 (3.62 - 3.99)
50-54	435597 (8.53%)	2294 (3.93%; 0.53%)	1785 (4.80%; 0.41%)	537 (2.40%; 0.12%)	3.22 (3.01 - 3.42)
55-59	404668 (7.92%)	1537 (2.63%; 0.38%)	1229 (3.31%; 0.30%)	327 (1.46%; 0.08%)	3.65 (3.39 - 3.91)
60-64	358974 (7.03%)	795 (1.36%; 0.22%)	623 (1.68%; 0.17%)	176 (0.79%; 0.05%)	3.40 (3.04 - 3.75)
65-69	332917 (6.52%)	438 (0.75%; 0.13%)	339 (0.91%; 0.10%)	107 (0.48%; 0.03%)	3.08 (2.61 - 3.54)
70-74	259560 (5.08%)	220 (0.38%; 0.08%)	184 (0.50%; 0.07%)	44 (0.20%; 0.02%)	4.21 (3.50 - 4.92)
≥75	454763 (8.90%)	144 (0.25%; 0.03%)	114 (0.31%; 0.03%)	33 (0.15%; 0.01%)	3.35 (2.53 - 4.18)
**Migratory background by age**					
Western					
<25 years	489965 (12.84%)	8090 (25.90%; 1.65%)	3778 (18.85%; 0.77%)	4451 (37.82%; 0.91%)	0.82 (0.73 - 0.92)
≥25 years	3325745 (87.16%)	23151 (74.10%; 0.70%)	16264 (81.15%; 0.49%)	7318 (62.18%; 0.22%)	2.15 (2.09 - 2.22)
Non-Western					
<25 years	268166 (20.75%)	8027 (29.60%; 2.99%)	3748 (21.91%; 1.40%)	4509 (42.44%; 1.68%)	0.81 (0.71 - 0.90)
≥25 years	1024045 (79.25%)	19088 (70.40%; 1.86%)	13360 (78.09%; 1.30%)	6116 (57.56%; 0.60%)	2.11 (2.04 - 2.18)
**Education level[5]**					
Low	1107656 (34.35%)	1107656 (27.38%; 1.30%)	9746 (29.75%; 0.88%)	4882 (23.52%; 0.44%)	1.94 (1.86 - 2.02)
Medium	1294141 (40.13%)	1294141 (47.17%; 1.91%)	14924 (45.56%; 1.15%)	10353 (49.87%; 0.80%)	1.40 (1.34 - 1.45)

High	822707 (25.51%)	822707 (25.45%; 1.62%)	8088 (24.69%; 0.98%)	5525 (26.61%; 0.67%)	1.41 (1.34 - 1.49)
Missing	1883417	5932	4392	1634	
**Area**					
**Degree of urbanisation**					
Very high (≥2500 addresses/km^2)	2414666 (47.29%)	41598 (71.30%; 1.72%)	25382 (68.34%; 1.05%)	17147 (76.58%; 0.71%)	1.43 (1.38 - 1.47)
High (1500 - 2500 addresses/km^2)	1548797 (30.33%)	12013 (20.59%; 0.78%)	8418 (22.66%; 0.54%)	3805 (16.99%; 0.25%)	2.18 (2.10 - 2.27)
Moderate (1000 - 1500 addresses/km^2)	690718 (13.53%)	2968 (5.09%; 0.43%)	2043 (5.50%; 0.30%)	956 (4.27%; 0.14%)	2.11 (1.94 - 2.27)
Low (500 - 1000 addresses/km^2)	295389 (5.78%)	1288 (2.21%; 0.44%)	971 (2.61%; 0.33%)	329 (1.47%; 0.11%)	2.72 (2.45 - 2.99)
Rural (<500 addresses/km^2)	156723 (3.07%)	476 (0.82%; 0.30%)	327 (0.88%; 0.21%)	153 (0.68%; 0.10%)	2.01 (1.60 - 2.42)
Missing	1628	13	9	4	
**Median household income**					
Highest (>€36,600)	1155750 (22.64%)	6304 (10.81%; 0.55%)	4402 (11.85%; 0.38%)	1990 (8.89%; 0.17%)	2.10 (1.98 - 2.21)
Upper middle (€28,400 - €36,600)	74093 (1.45%)	672 (1.15%; 0.91%)	387 (1.04%; 0.52%)	293 (1.31%; 0.40%)	1.24 (0.92 - 1.57)
Middle (€22,200 - €28,400)	1944899 (38.09%)	17985 (30.83%; 0.92%)	11690 (31.48%; 0.60%)	6650 (29.70%; 0.34%)	1.73 (1.66 - 1.80)
Lower middle (€16,800-€22,200)	1868444 (36.59%)	31996 (54.84%; 1.71%)	19834 (53.40%; 1.06%)	12848 (57.39%; 0.69%)	1.49 (1.44 - 1.54)
Lowest (<€16,800)	62792 (1.23%)	1384 (2.37%; 2.20%)	827 (2.23%; 1.32%)	608 (2.72%; 0.97%)	1.30 (1.08 - 1.53)
Missing	1943	15	10	5	

Abbreviations: CI, confidence interval; GP, general practitioner; km, kilometre; no, number; RR, rate ratio; SHC, sexual health centre; STI, sexually transmitted infection.

[1] The data underlying this table are the GP and SHC laboratory data, and the population register data (2015-2019).

[2] Proportion tested is based on the raw numbers. The mean HIV testing rates (number of HIV tests per 1,000 residents) are calculated over the study period of 5 year and corrected for data incompleteness. The number of tests by SHC was corrected with 1/0.88, considering the 88% match between SHC and population data. For each municipality, the number of tests by the GP was corrected by 1/coverage (municipality specific). The corrected SHC numbers are on average 13% higher (min. 9% - max. 15%), and the corrected GP numbers on average 9% higher (min. 4% - max. 13%).

[3] Comparison of HIV testing rate, with SHC as reference.

[4] Without Cape Verdean.

[5] The International Standard Classification of Education was used as basis (European Commission; available from: https://ec.europa.eu/eurostat/statistics-explained/index.php?title=International_Standard_Classification_of_Education_(ISCED)#:~:text=ISCED%201%3A%20Primary%20education,Post%2Dsecondary%20non%2Dtertiary%20education).

Supplementary Table 2. Providers-specific HIV diagnoses and incidence per 100,000 residents by year, 2015-2019[1]

	Total		GP		SHC		Hospital		Other[2]	
	**No. (%)**	**Incidence**	**No. (%)**	**Incidence**	**No. (%)**	**Incidence**	**No. (%)**	**Incidence**	**No. (%)**	**Incidence**
**Total**	539	10.55	156	3.05	145	2.84	154	3.01	84	1.64
2015	134 (24.90%)	13.33	39 (25.00%)	3.88	40 (27.60%)	3.98	32 (20.80%)	3.18	23 (27.40%)	2.29
2016	116 (21.50%)	11.45	26 (16.70%)	2.57	34 (23.40%)	3.36	32 (20.80%)	3.16	24 (28.60%)	2.37
2017	115 (21.30%)	11.26	38 (24.40%)	3.72	26 (17.90%)	2.55	37 (24.00%)	3.62	14 (16.70%)	1.37
2018	81 (15.00%)	7.86	24 (15.40%)	2.33	26 (17.90%)	2.52	18 (11.70%)	1.75	13 (15.50%)	1.26
2019	93 (17.30%)	8.95	29 (18.60%)	2.79	19 (13.10%)	1.83	35 (22.70%)	3.37	10 (11.90%)	0.96

Abbreviations: GP, general practitioner; no, number; SHC, sexual health centre.

[1] The data underlying this table are the SHM database which includes all people living with HIV that receive care, and the population register data for incidence estimates (2015-2019).

[2] The provider group Other consists of people diagnosed abroad, diagnosed at another location and diagnosed at an unknown location.
